# Grape-seed Polyphenols Play a Protective Role in Elastase-induced Abdominal Aortic Aneurysm in Mice

**DOI:** 10.1038/s41598-017-09674-4

**Published:** 2017-08-24

**Authors:** Chao Wang, Yunxia Wang, Maomao Yu, Cong Chen, Lu Xu, Yini Cao, Rong Qi

**Affiliations:** 1 Peking University Institute of Cardiovascular Sciences, Peking University Health Science Center, Peking University, Beijing, 100191 China; 2 Key Laboratory of Molecular Cardiovascular Sciences, Ministry of Education, Beijing, 100191 China; 3 Second School of Clinical Medicine, Peking University, Beijing, 100044 China; 4 Beijing Key Laboratory of Molecular Pharmaceutics and New Drug Delivery Systems, Beijing, 100191 China

**Keywords:** Pharmacodynamics, Valvular disease

## Abstract

Abdominal aortic aneurysm (AAA) is a kind of disease characterized by aortic dilation, whose pathogenesis is linked to inflammation. This study aimed to determine whether grape-seed polyphenols (GSP) has anti-AAA effects and what mechanism is involved, thus to find a way to prevent occurrence and inhibit expansion of small AAA. In our study, AAA was induced by incubating the abdominal aorta of the mice with elastase, and GSP was administrated to the mice by gavage at different doses beginning on the day of the AAA inducement. In *in vivo* experiments, 800 mg/kg GSP could significantly reduce the incidence of AAA, the dilatation of aorta and elastin degradation in media, and dramatically decrease macrophage infiltration and activation and expression of matrix metalloproteinase (MMP) −2 and MMP-9 in the aorta, compared to the AAA model group. Meanwhile, 400 mg/kg GSP could also but not completely inhibit the occurrence and development of AAA. In *in vitro* experiments, GSP dose-dependently inhibited mRNA expression of interleukin (IL)-1β, IL-6 and monocyte chemoattractant protein-1 (MCP-1), and significantly inhibited expression and activity of MMP-2 and MMP-9, thus prevented elastin from degradation. In conclusion, GSP showed great anti-AAA effects and its mechanisms were related to inhibition of inflammation.

## Introduction

Abdominal aortic aneurysm (AAA) is a cardiovascular disease characterized by aortic dilation that exceeds the normal diameter by 50% or exceeds 3 cm in the infra-renal region^1^. Age, male gender, family history of AAA, smoking and hypertension are all risk factors related to AAA^2^. According to the population-based ultrasound screening studies, the prevalence of AAA is 4–7% in male and 1–2% in female over the age of 65^3^. Most patients show no obvious clinical manifestations, but with the expanding of aneurysm diameter, the risk of aortic rupture gradually increased^4^. Once ruptured, the mortality is as high as 85–90%^5^, making AAA a serious threat to human health. However, no medicine in clinic can effectively cure the disease so far. Surgical repairs are recommended for patients with an aortic diameter over 5.5 cm, while for patients with an aortic diameter of 3.0–5.4 cm, which is defined as small AAA, nothing but keeping close surveillance can be applied to them^6^. Therefore, it is necessary to find medicinal health care to prevent the occurrence of AAA and efficiently inhibit the expansion of small AAA.

A series of studies have provided crucial findings about the pathogenesis of AAA, including infiltration of lymphocytes and macrophages into the aneurysmal lesion, synthesis and excretion of inflammatory mediators and protease (especially matrix metalloproteinases, MMPs), degradation of elastin and collagen in the media and adventitia, apoptosis of vascular smooth muscle cells (VSMCs) induced by inflammatory response, as well as destruction of arterial wall and expansion of aortic lumen^7^. Therefore, the pathological process of AAA formation is related to inflammation. Plant polyphenols are known for their beneficial effects on cardiovascular diseases, which are largely associated with their anti-oxidant and anti-inflammatory properties. In the past two decades, grape-seed polyphenols (GSP), including a large group of flavonoids, such as epicatechin, flavanols, anthocyanins, etc., have been demonstrated to have significant effects of anti-inflammation and anti-oxidant, which may contribute to the inhibition of AAA^8^. However, whether GSP help to prevent AAA remains unknown.

The present study was designed to verify the hypothesis that GSP has anti-AAA effects and the mechanism is related to its anti-inflammation effects. The anti-AAA effects of GSP were evaluated *in vivo* in an elastase-induced AAA mouse model and its anti-AAA mechanisms were explored *in vitro* in TNF-α stimulated VSMC.

## Results

### Effects of GSP on AAA prevention

**Figure 1:**
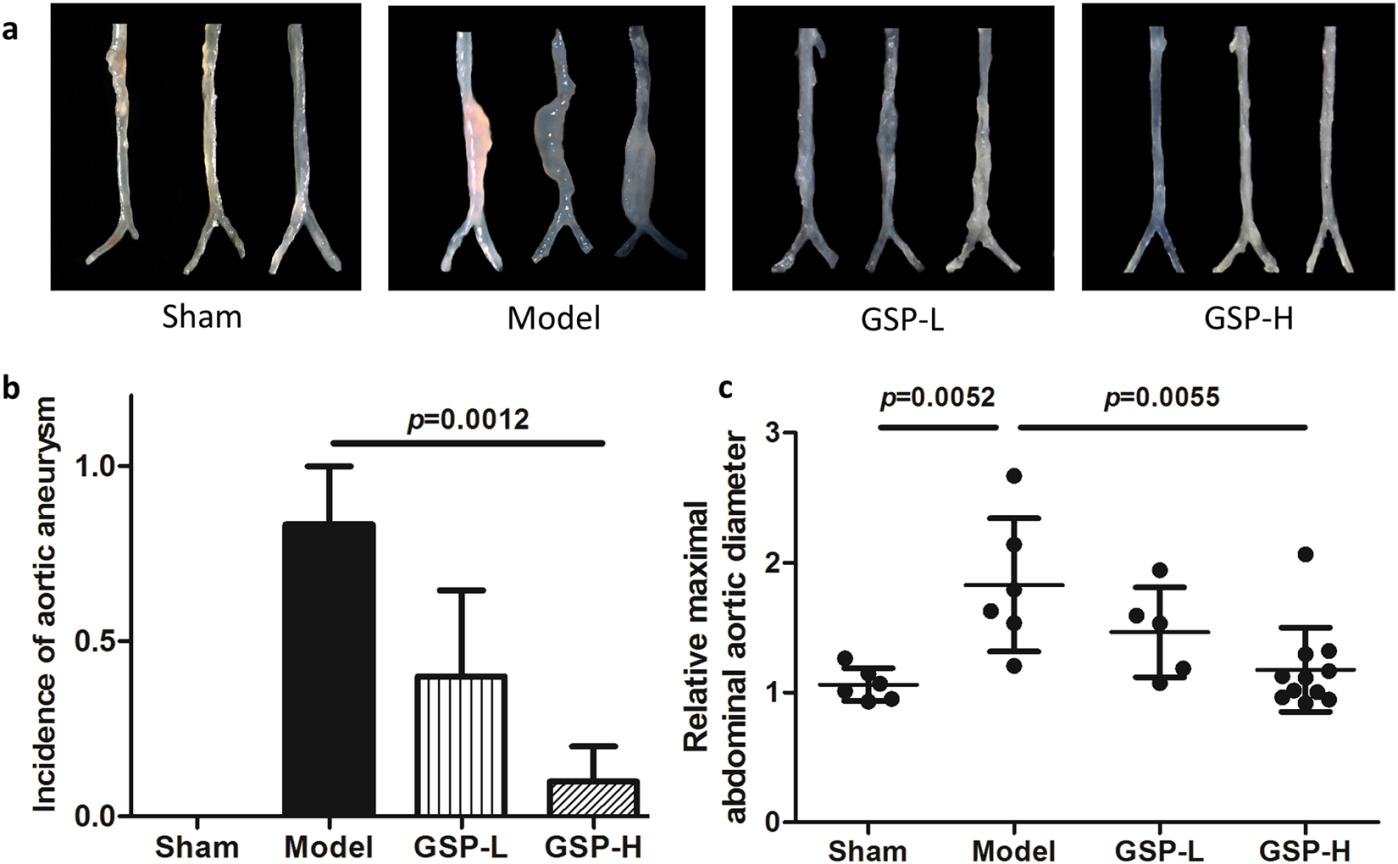
Effects of GSP on AAA prevention. Effects of GSP on (**a**) morphology, (**b**) incidence and (**c**) relative maximal abdominal aortic diameter of elastase-induced AAA in mice. n = 6, 6, 5, 11.

After induction by elastase for 14 days, mice in the AAA model group developed severe AAA, but GSP, especially at high dose, could completely inhibit the development of AAA (Fig. 1a). For quantitative analysis, we counted the incidence and the largest external diameters of artery in all mice groups and found that the high dose of GSP substantially reduced the incidence of AAA (9% *vs* 83%, *p* = 0.0012) and the dilation of infra-renal aortic lumen (1.18 *vs* 1.83, *p* = 0.0055), compared to the AAA model group. However, the low dose of GSP had no significant effects on AAA prevention (Fig. 1b,c).

### Pathological changes in mice arterial wall

**Figure 2:**
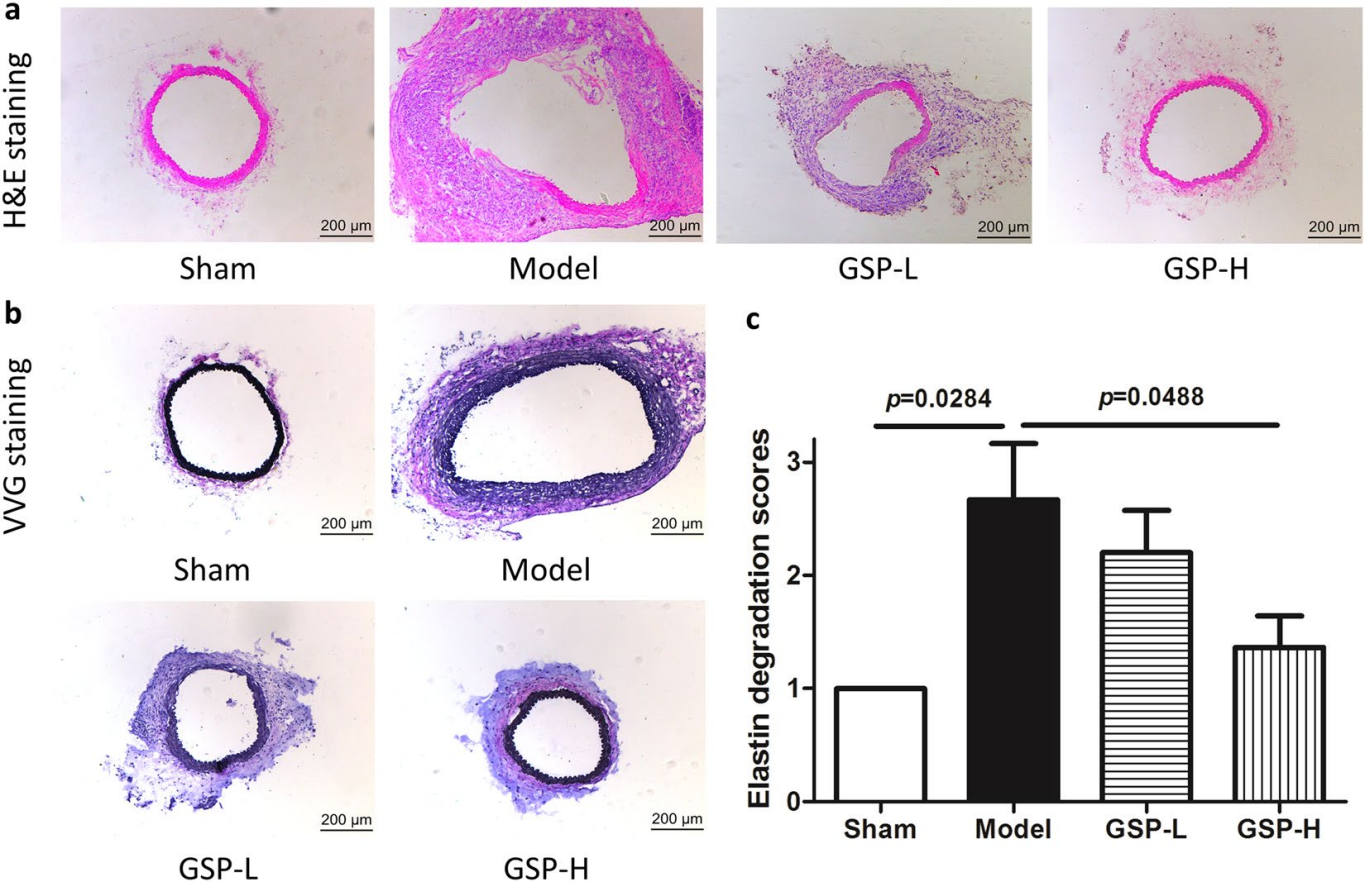
Preventive effects of GSP on elastase-induced AAA in mice. (**a**) H&E staining for aorta structure, (**b**) VVG staining and (**c**) quantification of elastin degradation. n = 6, 6, 5, 11.

Two weeks after the AAA induction, mice arteries from the model group presented severe dilation in the aortic lumen; also, flattening, fragmentation and degeneration of the elastic laminae in the medial layer, as well as thickening and remodeling in aortic adventitia could be seen from Hematoxylin-Eosin (H&E) staining and Verhoeff Van-Gieson (VVG) staining (Fig. 2a,b). Compared to the model group, the high dose of GSP could substantially reduce the elastin degradation in media (*p* = 0.0488), thus preserved the intact structure of aortic wall while the low dose of GSP had little effect (Fig. 2c).

### Effects of GSP on inflammatory infiltration in the arterial wall

**Figure 3:**
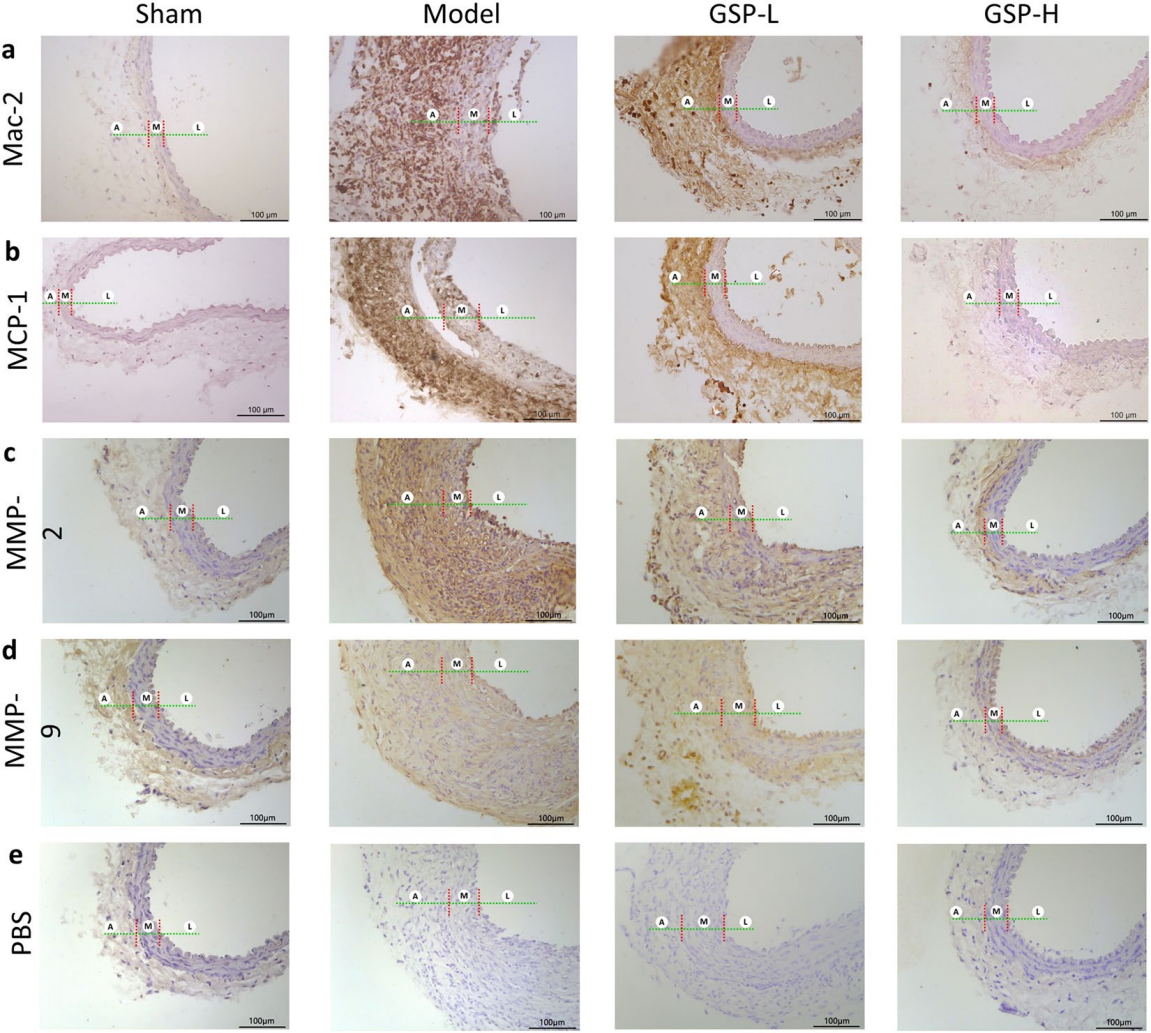
Effects of GSP administration at differnt doses on (**a**) macrophages infiltration, (**b**) MCP-1 expression, (**c**) MMP-2 expression, (**d**) MMP-9 expression and (**e**) representative negative controls of (**a**)~(**d**) in abdominal aorta of elastase-induced AAA mice. “L”, “M” and “A” were short for lumen, media and adventitia in the aorta, respectively.

Local inflammatory responses in the model group occurred with the AAA inducement and development, which were characterized by severe macrophage infiltration and over expression of Mac-2 and MCP-1. In comparison with the model group, treating the mice with GSP, especially with the high dose, significantly decreased macrophage infiltration and down-regulated mRNA expression of both Mac-2 and MCP-1 (Fig. 3a,b).

### Effects of GSP on the expression of MMP-2 and MMP-9 in the aorta

Fourteen days after elastase incubation, the expression of MMP-2 and MMP-9 in aorta was significantly increased in the model group. However, administration of GSP attenuated the expression of MMP-2 and MMP-9 in the aorta, especially in the high dose (Fig. 3c,d).

### Cytotoxicity of GSP on VSMC

**Figure 4:**
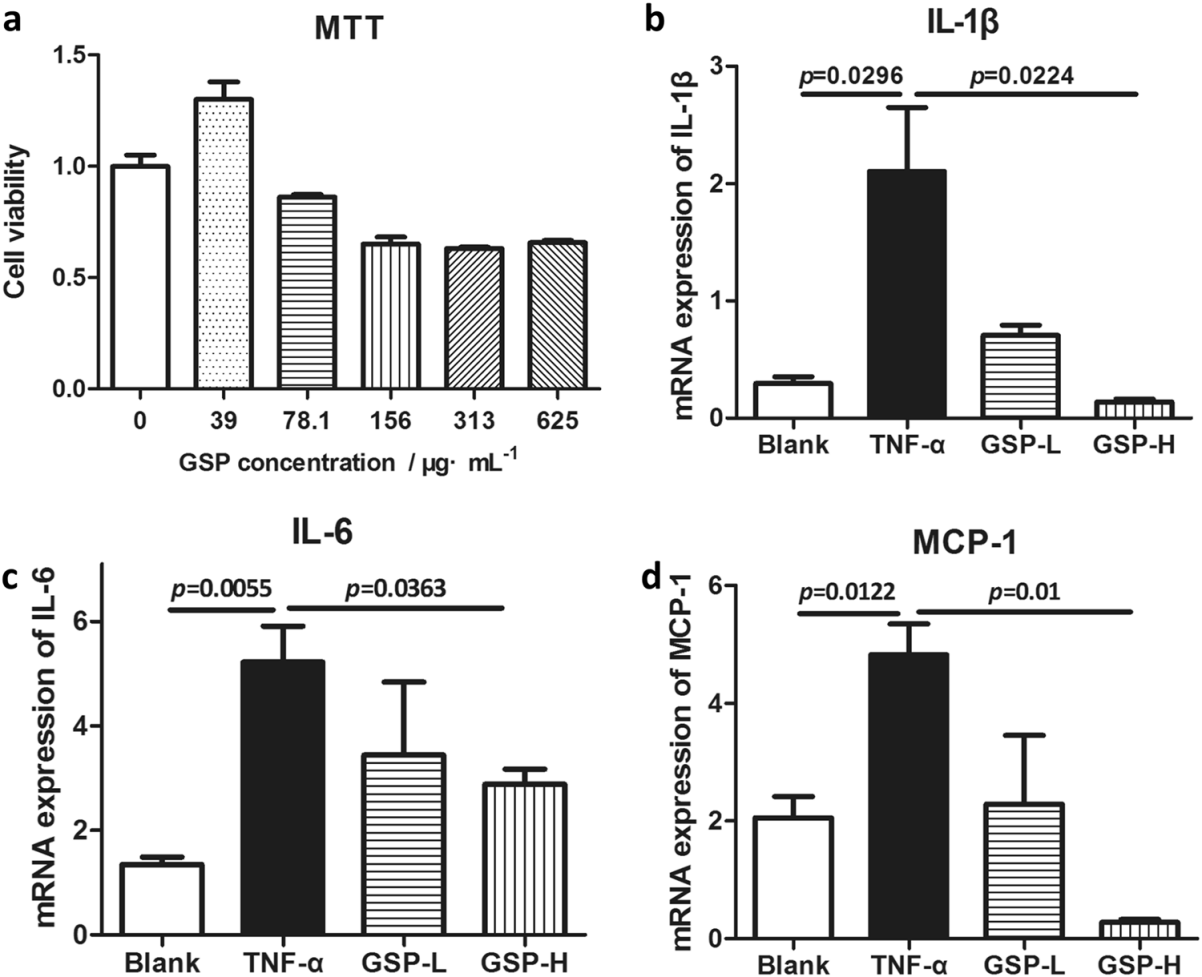
Effects of GSP on mRNA expression of pro-inflammatory cytokines in TNF-α stimulated VSMC. (**a**) cytotoxicity of GSP on rat VSMC (n = 4), (**b**–**d**) effects of GSP on mRNA expression level of (**b**) IL-1β, (**c**) IL-6, and (**d**) MCP-1 in VSMC stimulated by 100 ng/mL TNF-α. n = 3.

The results of MTT assay showed that GSP has obvious cytotoxicity on VSMCs (cell viabilities were less than 80% of the normal cell control) when its concentration was more than 156 μg/mL (Fig. 4a), but had no cytotoxic effects on VSMC at concentrations less than 78 μg/mL. Thus, GSP concentrations of 25 and 50 μg/mL were chosen for the following experiments.

### Effects of GSP on mRNA expression of pro-inflammatory cytokines

After treatment with TNF-α for 24 h, mRNA expression of pro-inflammatory cytokines of IL-1β (*p* = 0.0296), IL-6 (*p* = 0.0055) and MCP-1 (*p* = 0.0122) significantly increased compared to that of the untreated cells. However, the mRNA expression level of the above three cytokines were significantly decreased by treating the cells with GSP in a dose-dependent manner (Fig. 4b–d).

### Effects of GSP on MMPs and elastin

**Figure 5:**
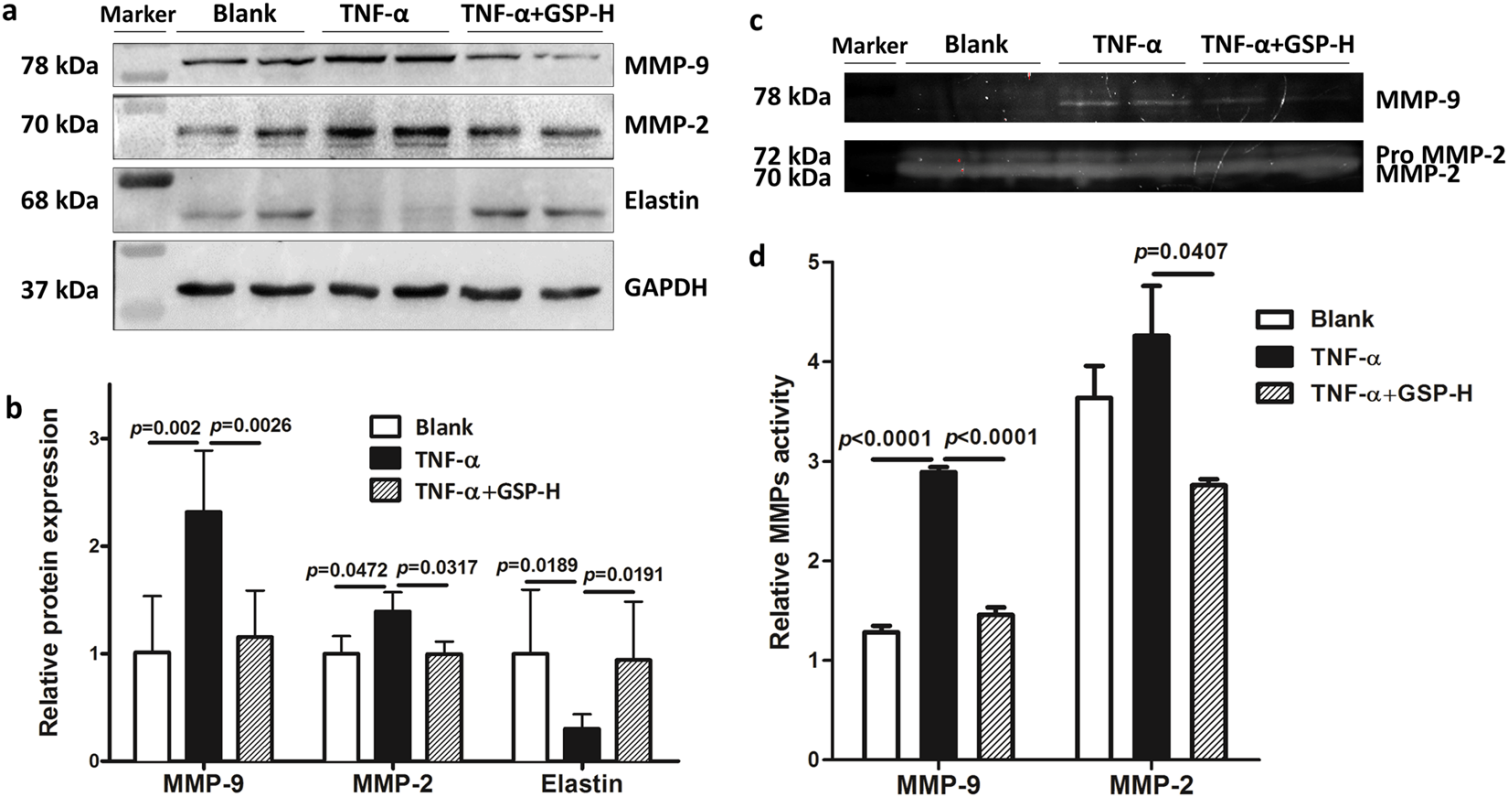
Effects of GSP on expression and activity of MMPs and expression of elastin in TNF-α stimulated VSMCs. (**a**) Protein expression level of MMP-2, MMP-9 and elastin, (**b**) quantifications of (**a**), (**c**) MMP-2 and MMP-9 enzymatic activity in the TNF-α-stimulated VSMCs and (**d**) quantifications of (**c**). n = 3.

The synthesis and secretion of MMP-2 and MMP-9 in VSMC was increased after the cells being treated with TNF-α, which further induced the degradation of elastin fibers. Results of western blot demonstrate that GSP down-regulated the expression of MMP-2 and MMP-9 and inhibited degradation of elastin (Fig. 5a,b), compared to the cells treated by TNF-α alone. Furthermore, activity of MMP-2 and MMP-9 was also inhibited by GSP, as shown in the results of gelatin zymography (Fig. 5c,d).

## Discussion

With a trend of leading a healthy life, plant-based diet is considered to be healthier than the diet laden with meat. Previous studies have demonstrated that plant-based diet could significantly reduce the incidence of coronary artery disease, which could largely be attributed to the key role of plant polyphenols^9^. Being a kind of active substances of the natural polyphenols, plant polyphenols extracted from dietary plants have attracted increasing attention due to its remarkable anti-inflammatory, anti-oxidant and further effects on anti-cardiovascular diseases^10, 11^. In this study, we confirmed the protective effects of GSP on AAA.

For *in vivo* study, AngII-induced aneurysm has proved to be an inflammation-driven model^12, 13^. However, in comparison with the AngII-induced AAA model, the elastase-induced AAA model has high morbidity and low mortality, and the aneurysm occurs in the infra-renal region while AngII-induced AAA occurs in the suprarenal abdominal aorta^14, 15^. In these aspects, the elastase model is more consistent with the situation of the AAA patients. So the anti-AAA effects of GSP were evaluated *in vivo* in an elastase-induced AAA mouse model. VSMCs are very important in keeping vascular structure and function, since they synthesize collagen, elastin, and other molecules of the extracellular matrix^16^. Inflammation can induce necrosis and apoptosis of VSMCs, and the matrix metalloproteinases secreted by VSMCs can degrade the extracellular matrix, all which lead to vessel damage^17^. Therefore, the anti-AAA mechanisms of GSP were explored *in vitro* in TNF-α stimulated VSMC.

In the AAA model group, infiltration of macrophages in aneurysmal tissue, which was a leading cause of AAA, was found severe. Macrophages can secrete inflammation mediators such as TNF-α, IL-1β and IL-6^18^, which was reported to over express in experimental AAA^11^, then recruit neutrophils, monocyte-macrophages and lymphocytes, and trigger inflammation responses, followed by apoptosis of VSMCs. Meanwhile, macrophages can secrete protease including collagenases and elastase, causing structure destruction together with reduction of elasticity and strength in the arterial wall, therefore resulting in the occurrence of AAA. Our study confirmed that GSP could reduce macrophage infiltration in local arterial wall, thus inhibiting the inflammatory responses and protecting integrity of arterial wall structure.

Chronic inflammation is one of the significant events in local arterial wall during AAA development. Preceding studies have provided a lot of evidence about the roles of cytokines like epidermal growth factor (EGF), IL-1β, IL-6 and IL-17 in regulating inflammatory cells in the development of AAA^19–21^. Inflammatory mediators can activate NF-kB^22^, ERK1/2 and p38 MAPK pathways^23^ in VSMCs, followed by enhancement in the secretion and activity of MMPs and apoptosis of VSMCs, leading to structural destruction of the arterial wall. Genetically knockout or specific antagonism of these inflammatory factors can prevent AAA formation induced by angiotensin II or elastase in mice^22, 23^. Our study suggests that GSP could reduce the expression of inflammatory mediators such as MCP-1 in *in vivo* and IL-1β and IL-6 in *in vitro*, resulting in the suppression of local arterial inflammation, thus preserved smooth muscle cells from apoptosis and inhibited damage of arterial structure.

Continuous lumen expansion is a typical manifestation of AAA. Local aortic dilation is derived from proteolytic disintegration of extracellular matrix in the arterial wall. Extracellular matrix degradation is mainly caused by two categories of proteases, matrix metalloproteinases and cathepsins^24–26^. Mice deficient in MMP-2 and MMP-9 had alleviated AAA induced by elastase or calcium chloride, showing the important role of MMPs in AAA development^27^. Therefore, MMPs inhibitors have been considered as a potential approach to prevent AAA progression^28^. Our results showed that GSP could reduce the protein expression and activity of MMP-2 and MMP-9. Therefore, GSP restrained the degradation of elastin and preserved the integrity of aorta wall structure and reduced the occurrence and development of AAA.

In this study, we confirmed the protective effects of GSP on AAA, and its anti-AAA mechanism was found to be related to the inhibition of local inflammation and the suppression the expression and activity of MMP-2 and MMP-9 in the abdominal aortic wall. Other studies have shown that polyphenols such as pentagalloyl glucose (PGG), epigallocatechin gallate (EGCG) and catechin, which are all plant polyphenols, could bind to monomeric tropoelastin and then enhance coacervation, aiding crosslinking of elastin by increasing lysyl oxidase (LOX) synthesis, which would repair the elastic lamina^29, 30^. Besides, in a previous study, GSP could prevent oxidative injury by modulating the expression of antioxidant enzyme systems^31^, and oxidative stress is also a pathological process involved in the formation of degenerative AAAs^1^. These might be other possible anti-AAA mechanisms of GSP and need to be clarified in our future studies.

This study indicated that oral administration of GSP could be a valuable potential for preventing the occurrence of AAA and inhibiting the expansion of small AAA. The specific composition and active monomers in GSP will be identified in our future studies.

## Methods

### Chemicals

GSP were kindly provided by Western Animal Husbandry Co., Ltd. (Xinjiang, China). TNF-α was obtained from Peprotech (Rocky Hill, USA). Fetal Bovine Serum (FBS), Dulbecco’s Modified Eagle’s Medium (DMEM), trypsin and Ethylene Diamine Tetraacetic Acid (EDTA) were obtained from GIBCO (Grand Island, USA). Tissue-Tek O.C.T. Compound was obtained from Sakura Finetek Japan Co., Ltd. (Tokyo, Japan). Antibodies of Mac-2, MCP-1, mouse anti-GAPDH primary antibody and horseradish peroxidase (HRP)-conjugated secondary antibodies were all purchased from Santa Cruz (Dallas, USA). Rabbit anti-MMP-2, rabbit anti-MMP-9 and rabbit anti-elastin primary antibodies were purchased from Abcam (Massachusetts, USA). Elastase, chloral hydrate, penicillin, streptomycin, MTT and all other reagents were purchased from Sigma-Aldrich (Beijing, China).

### Animal experiments

Healthy 8-week-old male C57BL/6 mice weighing 18–20 g were obtained from Department of Laboratory Animal Science, Peking University Health Science Center (Beijing, China). All mice were housed under the laboratory conditions of 12-hour light/dark cycle and temperature (25 ± 2 °C), and were given free access to food and water. All procedures involving animals were conformed to the Regulations for the Administration of Affairs Concerning Experimental Animals published by the State Science and Technology Commission of China and were approved by the Biomedical Ethics Committee of Peking University.

The mice were randomly assigned to sham group, model group, high dose GSP group (GSP-H) and low dose GSP group (GSP-L). Mice in the sham group and the model group were orally administered with 200 µL of 0.01 M phosphate buffered saline (PBS, pH = 7.4) once per day; the GSP-H group and the GSP-L group received GSP treatment at a dose of 800 mg/kg and 400 mg/kg of body weight, respectively, by oral administration once per day beginning on the same day of AAA inducement. All animals were fed with a standard chow diet.

Two weeks after the AAA inducement, all mice were weighed and sacrificed with overdose of pentobarbital anesthetization. The aortas were excised under a dissection microscope and photographed to measure the maximal and normal aortic diameters. AAA is defined when the dilation of the mice aortas exceeds the diameter of the normal mice aortas by 50%^32, 33^. The obtained tissues were fixed in 4% paraformaldehyde in PBS (pH 7.4) and embedded in Tissue-Tek O.C.T. Compound for histological analysis.

### AAA inducement

AAA was induced by local application of 1.5 U pancreatic elastase on the abdominal aortas of C57BL/6 mice. Briefly, the mice were anesthetized with intraperitoneal injection of sodium pentobarbital (60 mg/kg) and placed in a supine position on an animal operating table. After making a 1.5 cm midline incision in the abdominal wall, the abdominal aorta of the mice from infra-renal aorta to bifurcation of the aorta was isolated with blunt dissection. The separated abdominal aorta was then wrapped circumferentially with bilbulous paper soaked with 1.5 U pancreatic elastase (the sham group were treated by saline) for 40 min, after which the bilbulous paper was removed and the abdomen was sutured.

### Histological analysis

Specimens from the dilated aortas in the infra-renal region of the AAA mice or the corresponding aortas of the control or GSP-treated mice were embedded in Tissue-Tek O.C.T. Compound in liquid nitrogen, and then cut into 5 μm serial sections. H&E staining and VVG staining were used to analyze the morphology and evaluate elastin degradation of the mice aortas, respectively. Pathological score about elastin degradation in the aortas was executed double-blindly according to the following rules: score 1 for degradation less than 25%; score 2 for degradation ranging from 25% to 50%; score 3 for degradation ranging from 50% to 75%; score 4 for degradation greater than 75%^34^. Ten discontinuous sections in each specimen were used to quantify elastin degradation, and average score were obtained from the mean value of all specimens in each group.

Immunohistochemistry staining of Mac-2, MCP-1, MMP-2 and MMP-9 were used to observe the macrophage infiltration and inflammation as well as expression of MMP-2 and MMP-9 in the aorta. Briefly, the aortic slides were subjected to peroxidase quenching with 3% hydrogen peroxide, then incubated overnight with 100-fold diluted poly clonal primary antibodies at 4 °C. Subsequently, the slides were washed with PBS and then incubated with 500-fold diluted goat anti-rabbit or mice second antibodies conjugated with peroxidase at 37 °C for 60 min. After counterstained with hematoxylin, the slides were incubated with diaminobenzidine (DAB) peroxidase substrate to visualize peroxidase activity by light microscopy.

### Cell culture

Male Sprague Dawley (SD) rats weighting about 100 g were obtained from Department of Laboratory Animal Science, Peking University Health Science Center (Beijing, China).

Primary vascular smooth muscle cells (VSMC) were isolated from male SD rats weighing about 100 g according to the protocol described in the literature^35, 36^. Briefly, SD rats were anaesthetize by 10% chloral hydrate, and the whole aorta was excised and washed with 0.01 M PBS (containing 100 U/mL benzylpenicillin sodium and 100 mg/mL streptomycin sulfate). Following removal of intima and adventitia, arterial tissues was minced and further digested by collagenase type II for 2 h at 37 °C. At the end of digestion, the arteries were cut into 1 mm^2^ segments, and then transferred to a culture flask. After incubating in the bottom of flask for 6 h with Dulbecco’s modified Eagle’s medium (containing 20% fetal bovine serum and 100 U/mL benzylpenicillin sodium and 100 mg/mL streptomycin sulfate) at 37 °C (5% CO_2_), the flask was turned over and incubated for static culture for a week. After washed with 0.01 M PBS (pH = 7.4), the cells were trypsinized at 37 °C for 5 min. The cell passages of 5~7 were used in the following experiments.

### MTT assay for cell viability

The VSMC were cultured in 96-well plates (1 × 10^4^ cells/well) for 24 h. The cells were then treated with different concentrations of GSP for 24 h. After treatment, methylthiazolyl tetrazolium (MTT) was added to each well to a final concentration of 0.5 mg/mL and incubated for 4 h at 37 °C in a humidified incubator containing 5% CO_2_. Then 150 μL DMSO was used to dissolve formazan and absorbance at 490 nm was measured with an ELISA reader (No. 550, Hercules, California, USA).

### *In vitro* induction of AAA microenvironment and drug treatment

To establish an *in vitro* aneurysm microenvironment, 100 ng/mL TNF-α was applied to stimulate the VSMC for 48 h. In the meantime, the cells were treated with 25 or 50 μg/mL GSP. After incubated for 24 or 48 h, the cells were collected and RNA or proteins were extracted for PCR or western blot analysis. Parallel experiments were done in TNF-α treated only or non TNF-α treated normal cells (as blank).

### RT-PCR analysis

After 24-hour treatment, total RNA was extracted using Trizol Reagent (Molecular Research Center, USA) and cDNA synthesis was performed using the TransScript First-Strand cDNA Synthesis Super Mix (TransGen Biotech). EvaGreen qPCR MasterMix (abm, Canada) was used to evaluate mRNA expression levels according to the manufacturer’s instructions. The primers used for real-time PCR are shown in Table 1. And transcript levels were normalized to GAPDH (glyceraldehyde 3-phosphate dehydrogenase), which was used as an internal control.

**Table 1:** Primer sequences used in amplification PCR and semi-quantitative RT-PCR.

Primer	Sequence	
GAPDH	forward	TGATGACATCAAGAAGGTGGTGAAG
	reverse	TCCTTGGAGGCCATGTAGGCCAT
IL-1β	forward	GACTTCACCATGGAACCCGT
	reverse	GGAGACTGCCCATTCTCGAC
IL-6	forward	CCTTCTTGGGACTGATGT
	reverse	CTCTGGCTTTGTCTTTCT
MCP-1	forward	AATGAGTCGGCTGGAGAA
	reverse	GTGCTTGAGGTGGTTGTG

### Western blot analysis

After 48-hour treatment, the cells were collected and protein concentrations were quantified with a biscinchonic acid (BCA) kit (Pierce Biotechnology, Rockford, IL, USA). For western blot, 20 ng of protein was loaded in each well and resolved by 10% SDS-PAGE, and the protein bands were then electro-transferred onto a polyvinylidene difluoride membrane using the Bio-Rad MiniProtean II apparatus (Bio-Rad Laboratories, Carlsbad, CA, USA). The blots were subsequently incubated with anti-rabbit elastin (1:2000), anti-rabbit MMP-2 (1:1000), anti-rabbit MMP-9 (1:500), or anti-mouse GAPDH (1:5000) at 4 °C overnight followed by horseradish peroxidase (HRP)-conjugated secondary antibodies (1:5000) for 1 h and visualized with enhanced chemiluminescence system (Pierce Biotechnology, Rockford, IL, USA). GAPDH was used as an internal control for data normalization. All bands were quantified by Image J software.

### Gelatin zymography analysis

To determine the activity of MMP-2 and MMP-9, gelatin zymography was performed. Protein extracts (10 µg) were mixed with SDS buffer and electrophoresis was conducted (10% SDS-PAGE with 0.1% gelatin as substrate). Then the gels were washed with 2.5% Triton X-100 and incubated at 37 °C for 48 h with renaturing buffer followed by staining with coomassie brilliant blue (CBB) and destained with destaining solution containing 10% acetic acid and 40% methanol. Gels were scanned using Image-analyzer LAS-4000 (Fujifilm, Tokyo, Japan), and images were assessed by Image J.

### Statistical analysis

All data are presented as mean ± SD. *P* < 0.05 was considered to indicate statistically significant difference. Statistical significance of differences among the groups was analyzed by Student’s t-test, and elastin degradation scores between multiple groups was by one tailed Wilcoxon test. All statistical analyses were performed using GraphPad Prism for Windows (Version 4, San Diego, CA, USA).

## Electronic supplementary material

Effects of GSP on normal aorta.

## References

[1] Nordon, I. M., Hinchliffe, R. J., Loftus, I. M. & Thompson, M. M. Pathophysiology and epidemiology of abdominal aortic aneurysms. *Nat Rev Cardiol.* **8**, 92–102 (2011). 21079638 10.1038/nrcardio.2010.180

[2] Ahmed, R., Ghoorah, K. & Kunadian, V. Abdominal Aortic Aneurysms and Risk Factors for Adverse Events. *Cardiol Rev.* **24**, 88–93 (2016). 25580705 10.1097/CRD.0000000000000052

[3] Eckstein, H. H. *et al*. Ultrasonographic screening for the detection of abdominal aortic aneurysms. *Dtsch Arztebl Int.* **106**, 657–663 (2009). 19946430 10.3238/arztebl.2009.0657 PMC2780009

[4] Badger, S. A. *et al*. Surveillance strategies according to the rate of growth of small abdominal aortic aneurysms. *Vasc Med.* **16**, 415–421 (2011). 22128040 10.1177/1358863X11423971

[5] Filardo, G., Powell, J. T., Martinez, M. A. & Ballard, D. J. Surgery for small asymptomatic abdominal aortic aneurysms. *Cochrane Database Syst Rev.* **2**, CD001835 (2015). 10.1002/14651858.CD001835.pub4 PMC6464801 25927098

[6] Erbel, R. *et al*. ESC Guidelines on the diagnosis and treatment of aortic diseases: Document covering acute and chronic aortic diseases of the thoracic and abdominal aorta of the adult. The Task Force for the Diagnosis and Treatment of Aortic Diseases of the European Society of Cardiology (ESC). *Eur Heart J.* **35**, 2873–2926 (2014). 25173340 10.1093/eurheartj/ehu281

[7] Davis, F. M., Rateri, D. L. & Daugherty, A. Abdominal aortic aneurysm: novel mechanisms and therapies. *Curr Opin Cardiol.* **30**, 566–573 (2015). 26352243 10.1097/HCO.0000000000000216 PMC4624089

[8] Du, Y., Guo, H. & Lou, H. Grape seed polyphenols protect cardiac cells from apoptosis via induction of endogenous antioxidant enzymes. *J Agric Food Chem.* **55**, 1695–1701 (2007). 17295515 10.1021/jf063071b

[9] Tuso, P., Stoll, S. R. & Li, W. W. A plant-based diet, atherogenesis, and coronary artery disease prevention. *Perm J.* **19**, 62–67 (2015). 25431999 10.7812/TPP/14-036 PMC4315380

[10] Arafa, M. H., Mohammad, N. S., Atteia, H. H. & Abd-Elaziz, H. R. Protective effect of resveratrol against doxorubicin-induced cardiac toxicity and fibrosis in male experimental rats. *J Physiol Biochem.* **70**, 701–711 (2014). 24939721 10.1007/s13105-014-0339-y

[11] Leifert, W. R. & Abeywardena, M. Y. Cardioprotective actions of grape polyphenols. *Nutr Res.* **28**, 729–737 (2008). 19083481 10.1016/j.nutres.2008.08.007

[12] Wang, Y., Krishna, S. & Golledge, J. The calcium chloride-induced rodent model of abdominal aortic aneurysm. *Atherosclerosis.* **226**, 29–39 (2013). 23044097 10.1016/j.atherosclerosis.2012.09.010

[13] Tsai, S. H. *et al*. Zoledronate attenuates angiotensin II-induced abdominal aortic aneurysm through inactivation of Rho/ROCK-dependent JNK and NF-kappaB pathway. *Cardiovasc Res.* **100**, 501–510 (2013). 24225494 10.1093/cvr/cvt230

[14] Curci, J. A. Digging in the “soil” of the aorta to understand the growth of abdominal aortic aneurysms. *Vascular.* **17**(Suppl 1), S21–29 (2009). 19426606 10.2310/6670.2008.00085 PMC2714584

[15] Sun, Z. & Chaichana, T. Investigation of the hemodynamic effect of stent wires on renal arteries in patients with abdominal aortic aneurysms treated with suprarenal stent-grafts. *Cardiovasc Intervent Radiol.* **32**, 647–657 (2009). 19290574 10.1007/s00270-009-9539-1

[16] Robert, L. Elastin, past, present and future. *Pathol Biol (Paris).* **50**, 503–511 (2002). 12469520 10.1016/s0369-8114(02)00336-x

[17] Ailawadi, G. *et al*. Smooth muscle phenotypic modulation is an early event in aortic aneurysms. *J Thorac Cardiovasc Surg.* **138**, 1392–1399 (2009). 19931668 10.1016/j.jtcvs.2009.07.075 PMC2956879

[18] Michineau, S. *et al*. Chemokine (C-X-C motif) receptor 4 blockade by AMD3100 inhibits experimental abdominal aortic aneurysm expansion through anti-inflammatory effects. *Arterioscler Thromb Vasc Biol.* **34**, 1747–1755 (2014). 24876351 10.1161/ATVBAHA.114.303913

[19] Obama, T. *et al*. Epidermal growth factor receptor inhibitor protects against abdominal aortic aneurysm in a mouse model. *Clin Sci (Lond).* **128**, 559–565 (2015). 25531554 10.1042/CS20140696

[20] Johnston, W. F. *et al*. Genetic and pharmacologic disruption of interleukin-1beta signaling inhibits experimental aortic aneurysm formation. *Arterioscler Thromb Vasc Biol.* **33**, 294–304 (2013). 23288154 10.1161/ATVBAHA.112.300432 PMC3632435

[21] Wei, Z. *et al*. Inhibiting the Th17/IL-17A-related inflammatory responses with digoxin confers protection against experimental abdominal aortic aneurysm. *Arterioscler Thromb Vasc Biol.* **34**, 2429–2438 (2014). 25234817 10.1161/ATVBAHA.114.304435

[22] Yoshimura, K. *et al*. Inhibitory effect of statins on inflammation-related pathways in human abdominal aortic aneurysm tissue. *Int J Mol Sci.* **16**, 11213–11228 (2015). 25993292 10.3390/ijms160511213 PMC4463697

[23] Yang, C. Q. *et al*. MCP-1 stimulates MMP-9 expression via ERK 1/2 and p38 MAPK signaling pathways in human aortic smooth muscle cells. *Cell Physiol Biochem.* **34**, 266–276 (2014). 25033895 10.1159/000362997

[24] Kadoglou, N. P. & Liapis, C. D. Matrix metalloproteinases: contribution to pathogenesis, diagnosis, surveillance and treatment of abdominal aortic aneurysms. *Curr Med Res Opin.* **20**, 419–432 (2004). 15119978 10.1185/030079904125003143

[25] Lohoefer, F. *et al*. Quantitative expression and localization of cysteine and aspartic proteases in human abdominal aortic aneurysms. *Exp Mol Med.* **46**, e95 (2014). 24833013 10.1038/emm.2014.20 PMC3972792

[26] Lv, B. J., Lindholt, J. S., Wang, J., Cheng, X. & Shi, G. P. Plasma levels of cathepsins L, K, and V and risks of abdominal aortic aneurysms: a randomized population-based study. *Atherosclerosis.* **230**, 100–105 (2013). 23958260 10.1016/j.atherosclerosis.2013.05.018 PMC3752302

[27] Longo, G. M. *et al*. Matrix metalloproteinases 2 and 9 work in concert to produce aortic aneurysms. *J Clin Invest.* **110**, 625–632 (2002). 12208863 10.1172/JCI15334 PMC151106

[28] Davis, F. M., Rateri, D. L. & Daugherty, A. Mechanisms of aortic aneurysm formation: translating preclinical studies into clinical therapies. *Heart.* **100**, 1498–1505 (2014). 25060754 10.1136/heartjnl-2014-305648

[29] Isenburg, J. C., Simionescu, D. T., Starcher, B. C. & Vyavahare, N. R. Elastin stabilization for treatment of abdominal aortic aneurysms. *Circulation.* **115**, 1729–1737 (2007). 17372168 10.1161/CIRCULATIONAHA.106.672873

[30] Sinha, A., Nosoudi, N. & Vyavahare, N. Elasto-regenerative properties of polyphenols. *Biochem Biophys Res Commun.* **444**, 205–211 (2014). 24440697 10.1016/j.bbrc.2014.01.027 PMC3947410

[31] Puiggros, F. *et al*. Grape seed procyanidins prevent oxidative injury by modulating the expression of antioxidant enzyme systems. *J Agric Food Chem.* **53**, 6080–6086 (2005). 16028999 10.1021/jf050343m

[32] Daugherty, A., Manning, M. W. & Cassis, L. A. Angiotensin II promotes atherosclerotic lesions and aneurysms in apolipoprotein E-deficient mice. *J Clin Invest.* **105**, 1605–1612 (2000). 10841519 10.1172/JCI7818 PMC300846

[33] Yodoi, K. *et al*. Foxp3+ regulatory T cells play a protective role in angiotensin II-induced aortic aneurysm formation in mice. *Hypertension.* **65**, 889–895 (2015). 25601931 10.1161/HYPERTENSIONAHA.114.04934

[34] Satta, J. *et al*. Chronic inflammation and elastin degradation in abdominal aortic aneurysm disease: an immunohistochemical and electron microscopic study. *Eur J Vasc Endovasc Surg.* **15**, 313–319 (1998). 9610343 10.1016/s1078-5884(98)80034-8

[35] Wang, Q. *et al*. Receptor-interacting protein kinase 3 contributes to abdominal aortic aneurysms via smooth muscle cell necrosis and inflammation. *Circ Res.* **116**, 600–611 (2015). 25563840 10.1161/CIRCRESAHA.116.304899 PMC4329096

[36] Wang, H. J. *et al*. IP-10/CXCR3 Axis Promotes the Proliferation of Vascular Smooth Muscle Cells through ERK1/2/CREB Signaling Pathway. *Cell Biochem Biophys.* **75**, 139–147 (2017). 28111710 10.1007/s12013-017-0782-9

