# Supplementary material for: Grape-seed Polyphenols Play a Protective Role in Elastase-induced Abdominal Aortic Aneurysm in Mice
Source: Sci Rep. 2017 Aug 24;7:9402. doi: 10.1038/s41598-017-09674-4 (PMC5570906; doi:10.1038/s41598-017-09674-4)
Supplement: Supplementary file 1 — Effects of GSP on normal aorta. [file 41598_2017_9674_MOESM1_ESM.pdf]

Grape-seed Polyphenols Play a Protective Role in Elastase-induced Abdominal Aortic Aneurysm in Mice

Chao Wang [1, 2, 3, #], Yunxia Wang [1, 2, 4, #], Maomao Yu [1, 2, #], Cong Chen [1, 2, 4], Lu Xu [1, 2], Yini Cao [1, 2, 4], Rong Qi [1, 2, 4, *]

[1] Peking University Institute of Cardiovascular Sciences, Peking University Health Science Center, Peking University, Beijing, 100191, China

[2] Key Laboratory of Molecular Cardiovascular Sciences, Ministry of Education, Beijing, 100191, China

[3] Second School of Clinical Medicine, Peking University, Beijing, 100044, China

[4] Beijing Key Laboratory of Molecular Pharmaceutics and New Drug Delivery Systems, Beijing 100191, China

[#] These three authors contributed equally to this work.

[*] Corresponding author: ronaqi@bjmu.edu.cn

Supplemental Data

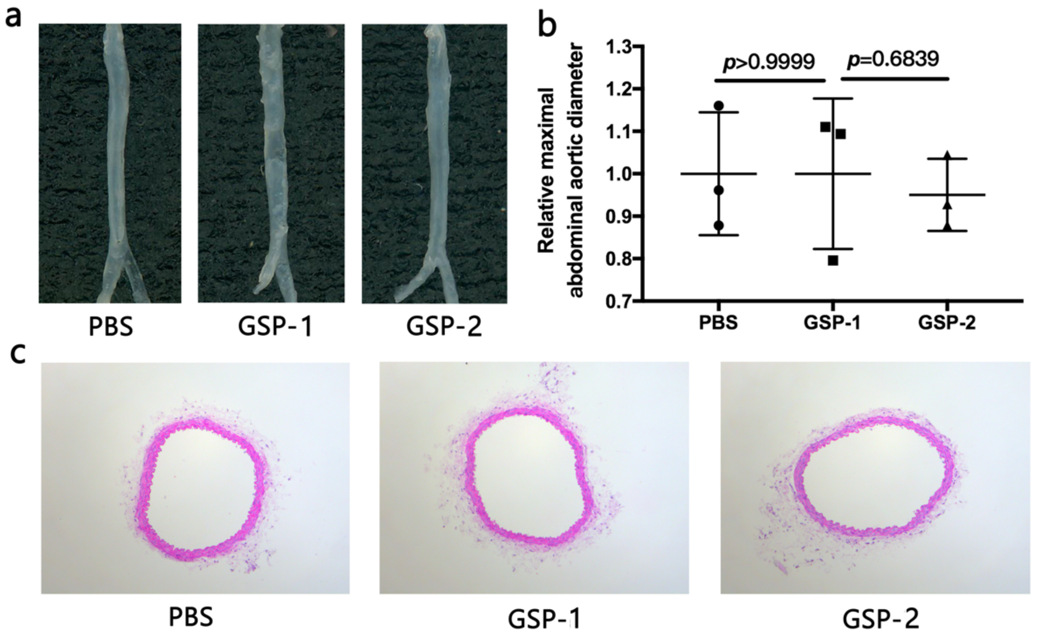

Fig. S1 Effects of GSP on normal aorta. Effects of GSP on (a) morphology, (b) maximal abdominal aortic diameter, (c) H&E staining for aorta structure, n=3. PBS, GSP-1 and GSP-2 were represented the mice groups administrated by 0.01M PBS, 800 mg/kg GSP and 1600 mg/kg GSP, respectively.
